# Nile Red staining of phytoplankton neutral lipids: species-specific fluorescence kinetics in various solvents

**DOI:** 10.1007/s10811-014-0404-5

**Published:** 2014-09-17

**Authors:** Katariina Natunen, Jukka Seppälä, Dagmar Schwenk, Heiko Rischer, Kristian Spilling, Timo Tamminen

**Affiliations:** 1 Marine Research Centre, Finnish Environment Institute, Erik Palménin aukio 1, P.O. Box 140, 00251 Helsinki, Finland; 2 VTT Technical Research Centre of Finland, Tietotie 2, 02044 Espoo, Finland

**Keywords:** Nile Red, Fluorescence kinetics, Phytoplankton, Neutral lipids, Solvents

## Abstract

Nile Red (NR) staining potentially offers a simple method for monitoring lipid accumulation in microalgal cultivation. However, variable staining efficiencies and methods have been reported. The effect of dimethyl sulfoxide (DMSO), ethylene glycol (EG) and glycerol on NR penetration with four different phytoplankton species representing different taxonomical groups was studied. Treatment with the solvents enhanced the NR fluorescence of the diatom *Phaeodactylum tricornutum* during kinetic fluorescence measurements, but high concentrations of solvents were needed. None of the solvents improved NR staining of the green alga *Chlorella pyrenoidosa* and *Scenedesmus obliquus*, which are known to be difficult to stain due to their thick and rigid cell walls. The naked *Isochrysis* sp. cells stained best without solvents. The results confirm that NR staining protocol needs to be optimized for each species.

## Introduction

Photosynthetic phytoplankton, also referred to as microalgae, are a potential feedstock for biofuels, especially because many species produce valuable neutral lipids (e.g. triacylglycerols) as storage products that can easily be converted to biodiesel (Scott et al. 2010; Williams and Laurens 2010). Some phytoplankton species have high maximum growth rates, doubling their biomass within hours during exponential growth (Chisti 2007). The amount of neutral lipids is typically low during exponential growth, but may start to accumulate rapidly when cell division slows down in the stationary growth phase. In many phytoplankton species, neutral lipids constitute 20–50 % of dry cell weight when the cells are cultivated under stressful conditions caused by chemical and/or physical factors, e.g. nutrient limitation, salinity or light intensity (Hu et al. 2008; Guschina and Harwood 2009). The accumulation of neutral lipids is thus highly dynamic and continuous, and rapid monitoring during phytoplankton cultivation is essential to find out why and when the lipids accumulate. In situ neutral lipid quantification methods, which usually require only small amounts of biomass, have proven to be particularly useful for this purpose (Gong and Jiang 2011).

The fluorescent benzophenoxazine dye Nile Red (NR) has been commonly used when studying the accumulation of neutral lipids in phytoplankton cultures based on a linear relationship between the amount of neutral lipids in cells and the fluorescence intensity of NR (Lee et al. 1998). NR is hydrophobic, and it is highly fluorescent in hydrophobic lipids as well as in all organic solvents (Greenspan et al. 1985).

Staining of phytoplankton neutral lipids with NR has been studied since the 1980s (Cooksey et al. 1987; Solomon et al. 1986; Greenspan et al. 1985). Cooksey et al. (1987) investigated the development of NR fluorescence as a function of time and found the shape of the resulting kinetic curve to vary between different species later supported by Elsey et al. (2007). Cooksey et al. (1987) suggested this to be due to different cellular permeabilities of NR and different lipid droplet sizes within the cells.

For the NR method to be feasible, the dye has to penetrate the algal cell wall to uniformly stain the intracellular neutral lipids, and poor permeability of NR is of course a great disadvantage (Chen et al. 2009; Huang et al. 2009). Chen et al. (2009) suggested that thick and rigid cell walls are the reason why the neutral lipids of many green algae species cannot be stained reliably with the original method by Cooksey et al. (1987). The solvent dimethyl sulfoxide (DMSO) turned out to be an effective carrier for NR (Chen et al. 2009), and its effectiveness was also demonstrated later (Kou et al. 2013; Wu et al. 2014; Doan and Obbard 2011). Doan and Obbard (2011) also successfully used glycerol to facilitate NR staining of green algae neutral lipids. Ethylene glycol (EG) had been used earlier as a solvent for NR staining of lipids in bivalve larvae (Castell and Mann 1994).

The objectives of this study were (i) to find out whether the NR penetration into phytoplankton cells and the subsequent staining of neutral lipids could be enhanced and ultimately standardized with the use of solvents in the case of phytoplankton species that have been proven difficult to stain, (ii) to evaluate whether the improved staining method developed by Chen et al. (2009) could be generalized to other algae types or (iii) whether the fluorescence kinetic parameters need to be evaluated separately for each species and growth phase, as suggested by Cooksey et al. (1987).

## Materials and methods

### Cell cultivation and monitoring of growth

The phytoplankton species chosen for the experiments were the eukaryotic, brackish water species *Phaeodactylum tricornutum* TV335 (Bacillariophyceae; fusiform cell type), *Chlorella pyrenoidosa* TV216 (Chlorophyceae) and *Scenedesmus obliquus* TVK-SOB-1 (Chlorophyceae) isolated from the Baltic Sea and *Isochrysis* sp. CCAP 927/12 (Haptophyceae) isolated from brackish water in England. All the selected species are promising for lipid production (Schwenk et al. 2013), but during our initial tests, they have shown variable NR fluorescence properties.

The phytoplankton species were cultivated in 2 L polycarbonate bottles using 1.5 L of modified f/2 medium (Guillard 1975) (salinity 6 g L^−1^) that had been adjusted to the weight ratios of N/P = 1.8 and N/Si = 0.25 so that the cells would be nitrogen limited in the stationary growth phase. Only the medium for the diatom *P. tricornutum* contained silicate. The batch cultures were continuously bubbled with sterile air at 22 ± 1 °C under 250–290 μmol photons m^−2^ s^−1^ irradiation in a 16:8 h light/dark cycle.

Growth was monitored by almost daily measurements of cell concentration (cells mL^-1^) with a FlowCAM particle analyser (Fluid Imaging Technologies, Inc.). NR fluorescence (excitation 530 nm/emission 580 nm) was measured with a Varian Cary Eclipse spectrofluorometer. The culture sample (2.7 mL) was stained with 12 μL of a 0.25 mg mL^−1^ stock solution of NR powder (Sigma, CAS number: 7385-67-3, Catalogue number: 72485) dissolved in acetone so that the final NR concentration in the sample was 1 μg mL^−1^. Cell wall penetration of the dye was enhanced by adding 0.3 mL of DMSO [Sigma-Aldrich, final concentration 10 % (*v*/*v*)]. For lipid accumulation monitoring purposes and as a starting point for the study, a 10-min NR staining time in darkness was used, after which, the sample cuvette was placed into the spectrofluorometer. The samples for cell count and fluorescence measurements were taken at the same time in the morning on each experimental day.

### Sampling for fluorescence kinetics in various solvents

**Fig. 1:**
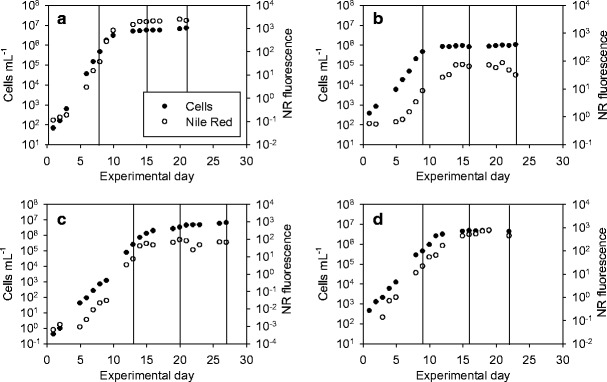
Development of cell concentration and NR fluorescence during the experiment: *P. tricornutum* (**a**), *S. obliquus* (**b**), *C. pyrenoidosa* (**c**) and *Isochrysis* sp. (**d**). Sampling days are indicated with *vertical lines*

Samples were taken in the exponential (Exp) and stationary growth phases (Stat 1). The stationary growth phase sampling was replicated after 6–7 days (Stat 2) (Fig. 1). *P. tricornutum* was cultivated for a total of 21 days (sampling on days 8, 15 and 21; Fig. 1a), *S. obliquus* for 23 days (sampling on days 9, 16 and 23; Fig. 1b), *C. pyrenoidosa* for 27 days (sampling on days 13, 20 and 27; Fig. 1c) and *Isochrysis* sp. for 22 days (sampling on days 9, 16 and 22; Fig. 1d).

On each sampling day, the effect of different final concentrations of DMSO (5, 10, 20 and 30 % *v*/*v*), EG (Riedel-de Haën; 1, 5, 10 and 20 % *v*/*v*) and glycerol (Normapur; 5, 10, 15 and 20 % *v*/*v*) on NR penetration into the cells was followed by kinetic fluorescence measurements. The final volume of each sample was 3 mL, containing 2 mL of phytoplankton culture, 12 μL of 0.25 mg mL^−1^ NR (final concentration 1 μg mL^−1^) and 1 mL of MQ water and solvent in such ratio that the desired solvent concentration (% *v*/*v*) was obtained (e.g. for 10 % *v*/*v* DMSO, 0.3 mL of DMSO and 0.7 mL of MQ water were added). For every measurement series, one phytoplankton sample without solvent (0 % *v*/*v*) was measured as a reference for solvent effects on the kinetic behaviour of the NR fluorescence. In addition, for each kinetic measurement, a blank value without the phytoplankton culture was measured so that the NR fluorescence in water and in each concentration of the solvents (i.e. background fluorescence) could be excluded from the kinetically measured fluorescence values.

NR fluorescence of the samples was recorded every 30 s for a total of 20 min in darkness inside the spectrofluorometer. For each sampling point, 13 such kinetic measurements were done (blank + 3 solvents at 4 concentrations), taking up to 5–6 h. Thus, replication of single kinetic measurements was not feasible. NR and the solvent (in MQ water) were added to the sample after 1 min of fluorescence measurement. The measurement points before the additions represent the baseline fluorescence without NR for the kinetic curve. The samples were continuously mixed during the measurement with a magnetic stirrer to keep the cells in suspension. The obtained NR fluorescence values were divided by the total lipid concentration (mg L^−1^) of the cultures in every growth phase to obtain lipid-specific NR fluorescence values.

#### Analyses

On each sampling day, samples for cellular particulate organic carbon and nitrogen (POC and PON, respectively, μg L^−1^), dry weight (DW, mg L^−1^) and total lipids (% *w*/*w*) were taken.

For POC and PON analyses, duplicate samples were filtered onto acid-washed and precombusted Whatman GF/F glass fibre filters and dried at room temperature in darkness. The samples were analyzed with a Roboprep/Tracermass mass spectrometer (Europa Scientific, UK). From the results, C/N weight ratios were obtained.

For DW determinations, duplicate samples were filtered onto precombusted (450 °C, 4 h) and preweighed Whatman GF/F glass fibre filters and stored at room temperature in darkness. The filters with the samples were weighed after drying them at 60 °C overnight.

For total lipid analyses, cells were harvested (i.e. 0.1‒0.4 mg of wet weight) by centrifugation, and the pellets were stored at −80 °C prior to the analyses. The total lipid content (% mg/mg DW) of the samples was determined by disrupting the cells and extracting the fatty acids with chloroform. The fatty acids were determined using transmethylation and gas chromatography (GC) with flame ionization detector (FID) according to Spilling et al. (2013). The total lipid concentrations (mg L^−1^) were obtained by multiplying the total lipid contents (% *w*/*w*) by the analyzed DW (mg L^−1^) values.

## Results and discussion

### Algae growth and lipid accumulation

The results of the daily measurements of cell concentration with FlowCAM and NR fluorescence are presented in Fig. 1; sampling days for kinetic NR fluorescence measurements and analyses are indicated with lines.

The intracellular C/N weight ratio increased from the first sample to the second in every species, as did the total lipid content (% *w*/*w*), confirming that the cultures had entered stationary growth phase when the second sample was taken (Table 1). In the exponential growth phase, the C/N ratio varied between 4.9 and 6.2 close to the Redfield ratio of 6.6. Towards the stationary growth phase, there was a 3- to 7-fold increase in the C/N ratio. Similar C/N results for *P. tricornutum* TV335, *S. obliquus* TVK-SOB-1 and *Isochrysis* sp. CCAP 927/12, cultivated in comparable conditions, were observed by Schwenk et al. (2013). Shifrin and Chisholm (1981) observed corresponding C/N values in the exponential growth phase for four different UTEX strains of *C. pyrenoidosa* cultivated in fresh water, but compared to the stationary growth phase, the increase in the C/N was lower than in this study.

**Table 1:** Cellular C/N weight ratio and total lipids (% *w*/*w*) in the three growth phases of *P. tricornutum*, *S. obliquus*, *C. pyrenoidosa* and *Isochrysis* sp.

Species	C/N weight ratio			Lipids (% *w*/*w*)		
	Exp	Stat 1	Stat 2	Exp	Stat 1	Stat 2
*P. tricornutum*	6.0	30.6	37.8	3.0	31.5	38.3
*S. obliquus*	4.8	18.2	23.0	5.2	25.8	36.6
*C. pyrenoidosa*	4.9	26.5	33.3	5.7	25.1	33.2
*Isochrysis* sp.	6.2	16.1	17.2	10.1	15.8	23.6

The total lipid contents varied in the exponential growth phase between 3.0 and 10.1 % (*w*/*w*) (Table 1). The largest (13-fold) increase in total lipid content between the exponential and stationary growth phases was observed in *P. tricornutum* and the smallest, 2-fold, in *Isochrysis* sp. Corresponding levels of total lipids in the stationary growth phase have been observed for *P. tricornutum*, *S. obliquus* and *Isochrysis* sp. (Schwenk et al. 2013) and *C. pyrenoidosa* (Shifrin and Chisholm 1981).

### Effect of solvents on Nile Red fluorescence kinetics

**Fig. 2:**
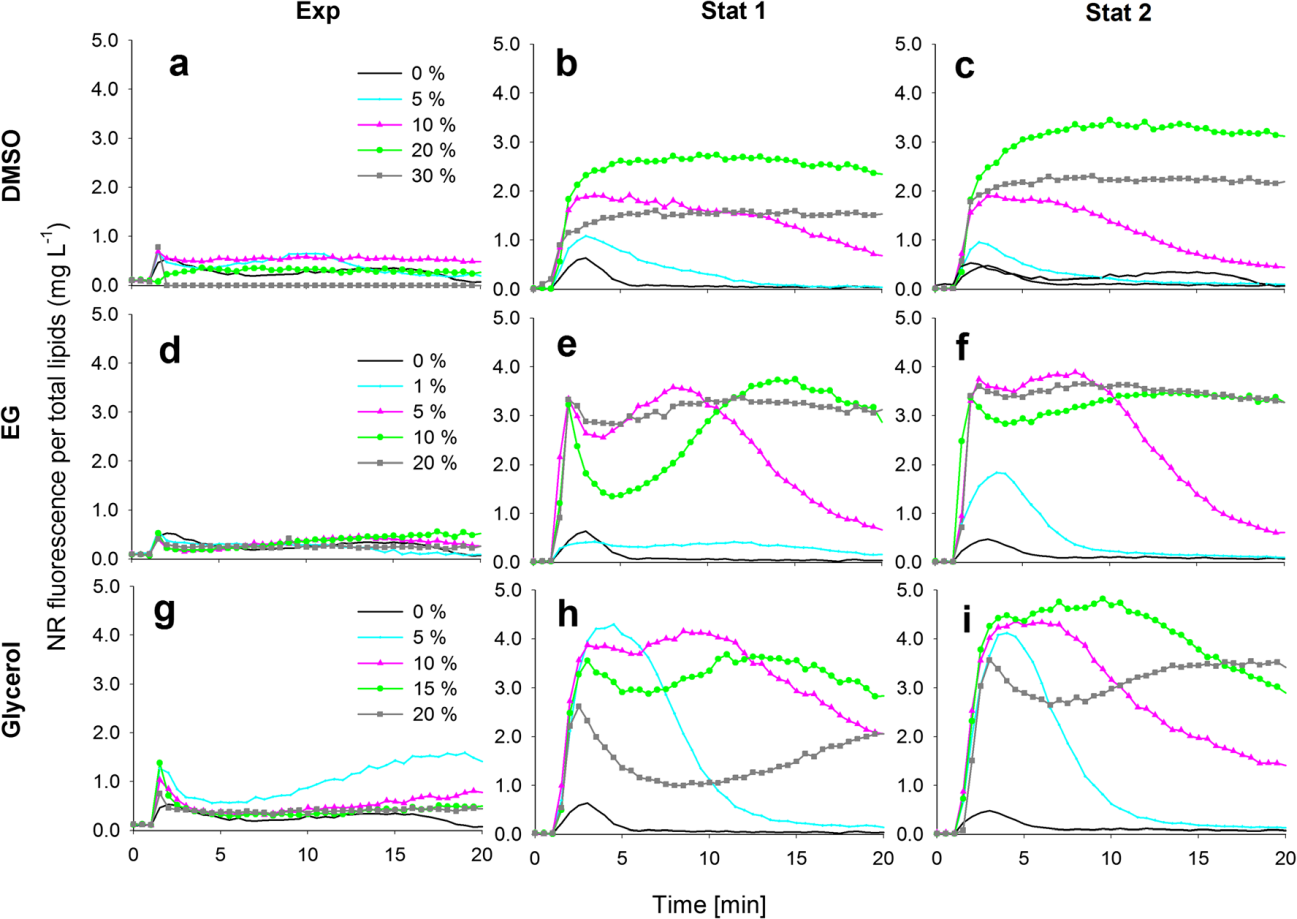
The effect of dimethyl sulfoxide (*DMSO*), ethylene glycol (*EG*) and glycerol on the NR staining of *P. tricornutum* cells in exponential and stationary (1 and 2) growth phases. The kinetic NR fluorescence data is divided by the total lipid concentration in the culture (mg L^−1^)

**Fig. 3:**
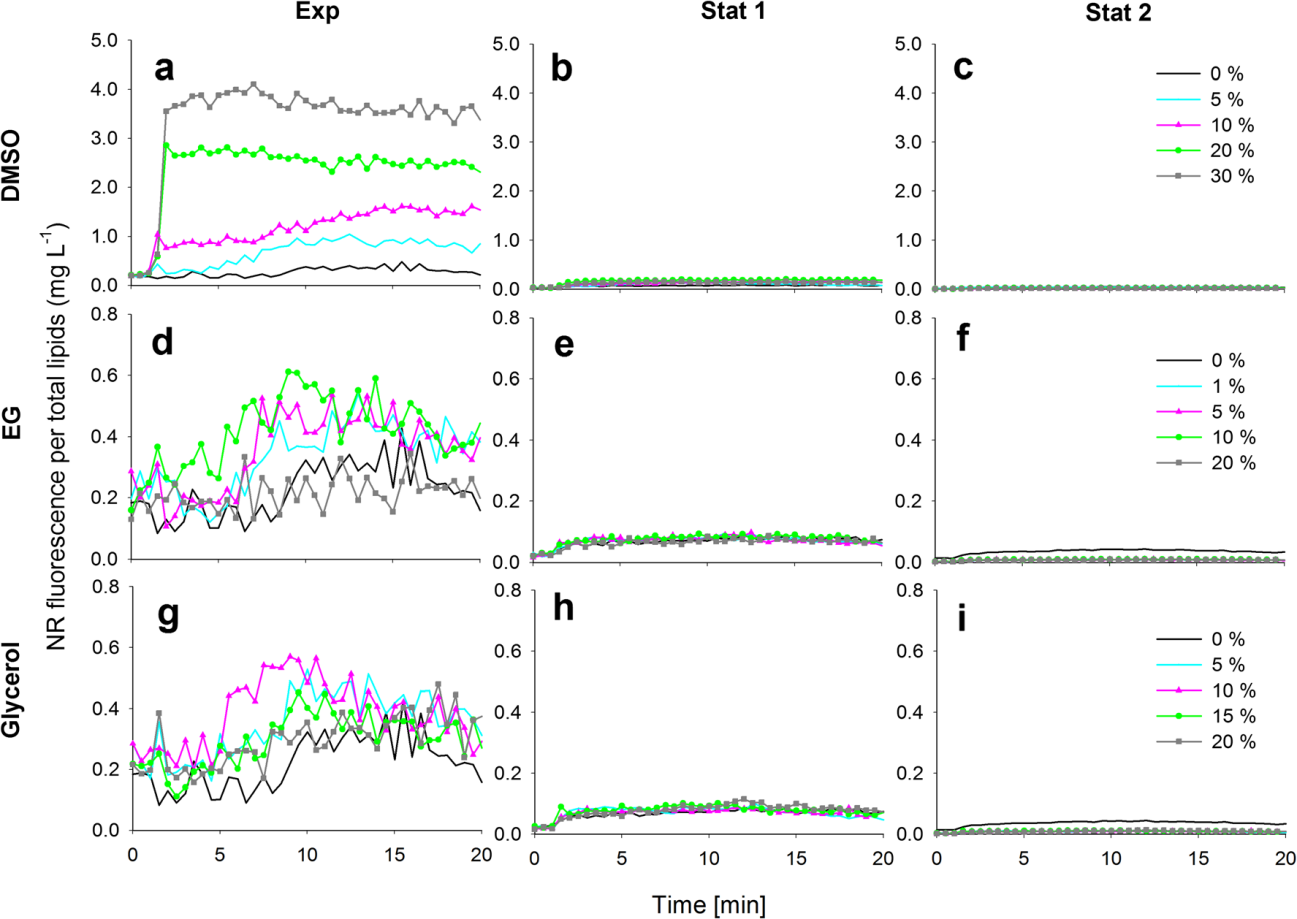
The effect of dimethyl sulfoxide (*DMSO*), ethylene glycol (*EG*) and glycerol on the NR staining of *S. obliquus* cells in exponential and stationary (1 and 2) growth phases. The kinetic NR fluorescence data is divided by the total lipid concentration in the culture (mg L^−1^). Different *y*-axis scales

**Fig. 4:**
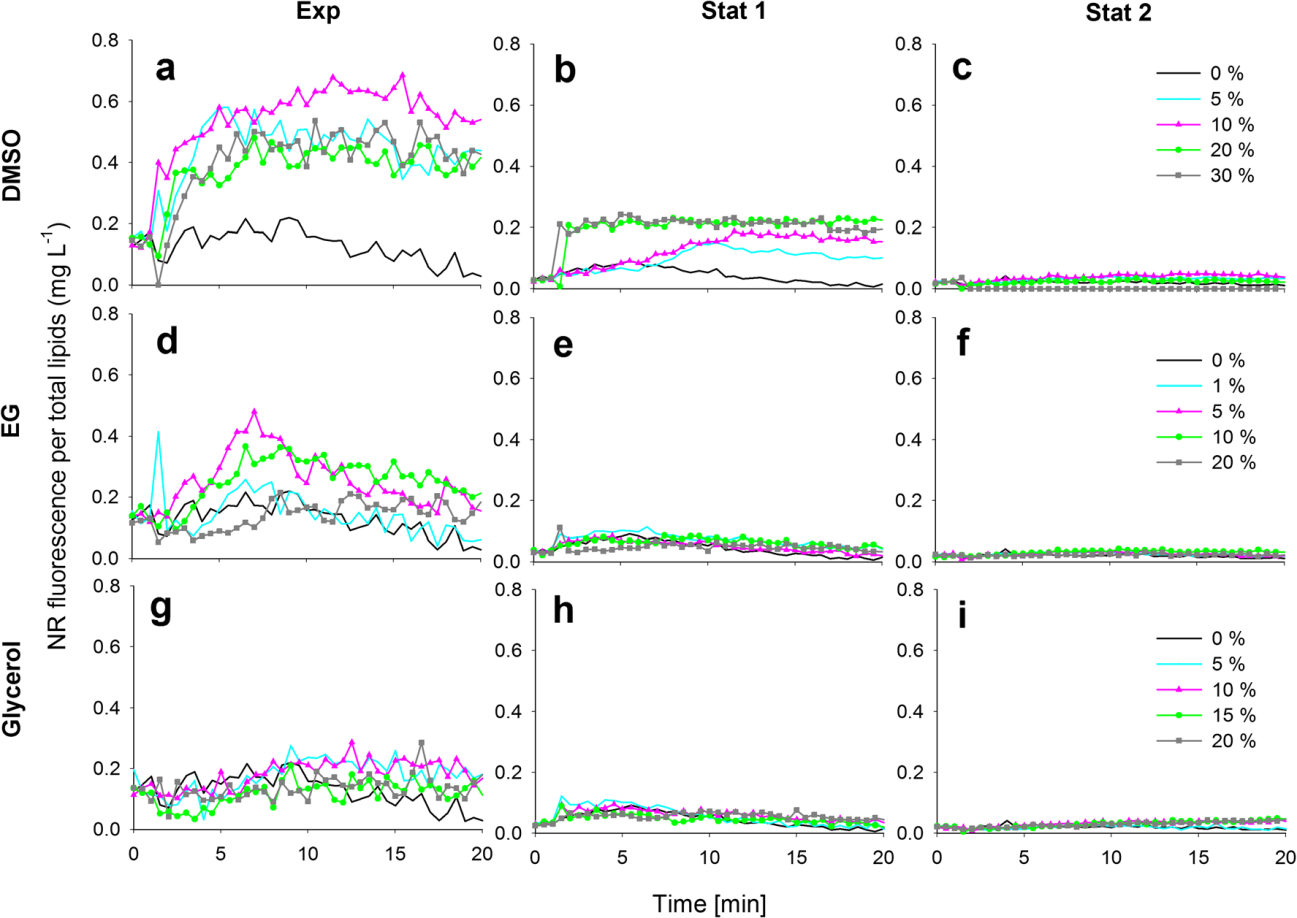
The effect of dimethyl sulfoxide (*DMSO*), ethylene glycol (*EG*) and glycerol on the NR staining of *C. pyrenoidosa* cells in exponential and stationary (1 and 2) growth phases. The kinetic NR fluorescence data is divided by the total lipid concentration in the culture (mg L^−1^)

**Fig. 5:**
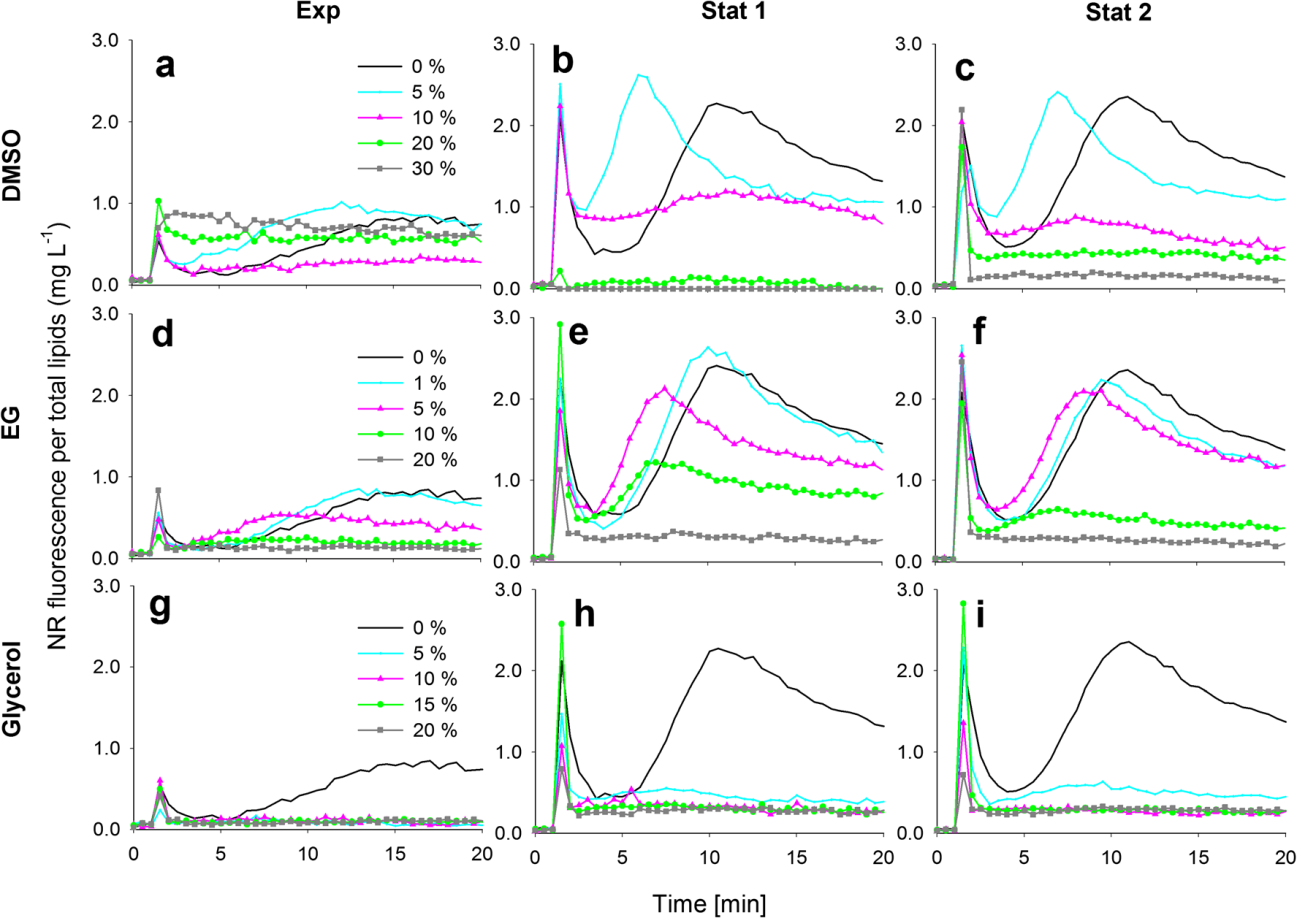
The effect of dimethyl sulfoxide (*DMSO*), ethylene glycol (*EG*) and glycerol on the NR staining of *Isochrysis* sp. cells in exponential and stationary (1 and 2) growth phases. The kinetic NR fluorescence data is divided by the total lipid concentration in the culture (mg L^−1^)

The effect of solvents DMSO, EG and glycerol on the NR fluorescence kinetics per total lipids (mg L^−1^) in each growth phase for *P. tricornutum*, *S. obliquus*, *C. pyrenoidosa* and *Isochrysis* sp. are presented in Figs. 2, 3, 4 and 5. It needs to be noted that in the exponential growth phase, the relative portion of neutral lipids in total lipids is much lower than in the stationary growth phase, where the NR-detectable neutral lipids start to accumulate (for a review, see Williams and Laurens 2010).

#### Nile Red fluorescence kinetics without solvents

In Figs. 2, 3, 4 and 5, the black 0 % (*v*/*v*) curves represent the lipid-specific NR fluorescence for each species and growth phase.

The characteristic NR fluorescence kinetic curve in *P. tricornutum* included a transient peak at ca. 2–3 min of staining, especially in the stationary growth phase (Fig. 2). Although lipids accumulated in *P. tricornutum* cells (Table 1), the lipid-specific NR fluorescence remained low, indicating that the dye was not able to penetrate the cell wall of the fusiform *P. tricornutum* strain without the aid of solvents.

A modest increase in the lipid-specific NR fluorescence at 10 min was observed in the exponential growth phase of *S. obliquus*, but in the stationary growth phase, the lipid-specific NR fluorescence was lower than in the exponential growth phase, although more lipids had been accumulated (Fig. 2, Table 1). The decrease in the lipid-specific NR fluorescence from exponential to stationary growth phases was also observed with *C. pyrenoidosa* (Fig. 3, Table 1).

In the case of *Isochrysis* sp., after the peak caused by the NR addition at 1 min, the NR fluorescence without solvents decreased, but started to increase again in exponential and stationary growth phases after 5 min (Fig. 5). Unlike the other species tested, the lipids of *Isochrysis* sp. were stainable without solvents.

#### DMSO

In the exponential growth phase of *P. tricornutum* (Fig. 2a), the NR fluorescence per total lipids was overall low because only a very small portion of the total lipids were neutral storage lipids. With DMSO, a modest solvent effect was observed. In the stationary growth phase (Fig. 2b, c), 20 % (*v*/*v*) DMSO produced the highest and most stable values.

DMSO had an influence on NR staining in the exponential growth phase of *S. obliquus* (Fig. 3a). The lipid-specific NR fluorescence values increased with increasing DMSO concentrations, and maximum fluorescence was achieved with the highest DMSO concentration (30 % *v*/*v*). In the stationary growth phase (Fig. 3b, c), however, the cells did not stain with any concentration of DMSO.

Also, in the case of *C. pyrenoidosa* (Fig. 4a), DMSO increased NR fluorescence compared to the fluorescence obtained without DMSO (0 % *v*/*v*) in the exponential growth phase. On the first stationary growth phase sampling day (Fig. 4b), DMSO increased the fluorescence slightly. The highest and most stable fluorescence values were obtained with 20 and 30 % (*v*/*v*) DMSO. On the second stationary growth phase sampling day (Fig. 4c), DMSO had no effect on the NR fluorescence values.

In the exponential growth phase of *Isochrysis* sp. (Fig. 5a), the 5 % (*v*/*v*) DMSO produced the highest fluorescence, but the signal was not stable, which was the case without DMSO as well. The 20 and 30 % (*v*/*v*) DMSO concentrations produced stable signals instead, but the intensities were not as high as with the 5 % (*v*/*v*) concentration and without DMSO. In the stationary growth phase (Fig. 5b, c), the fluorescence intensities decreased with increasing DMSO concentrations. The most stable signal was produced with 10 % (*v*/*v*) DMSO, but the intensity was lower than what was produced with 0 and 5 % (*v*/*v*) DMSO.

#### EG

EG had no effect in the exponential growth phase of *P. tricornutum* (Fig. *2*d). In the stationary growth phase (Fig. 2e, f), a stable signal was achieved with the highest EG concentration of 20 % (*v*/*v*). EG did not have an effect on the NR penetration into *S. obliquus* on any sampling day (Fig. 3d, e and f), and the same was observed with *C. pyrenoidosa* (Fig. 4d, e and f). In the exponential growth phase of *Isochrysis* sp. (Fig. 5d), only the lowest (1 % *v*/*v*) concentration of EG produced a high fluorescence that was not, however, higher or more stable than the fluorescence produced without EG. In the stationary growth phase (Fig. 5e, f), the lowest EG concentration (1 % *v*/*v*) and the solution without solvent produced a similar fluorescence curve and equally high values. Higher concentrations only decreased the fluorescence.

#### Glycerol

Glycerol at 5 % (*v*/*v*)produced a modest solvent effect in the exponential growth phase of *P. tricornutum* (Fig. 2g). In the stationary growth phase (Fig. 2h, i), the most stable signals were obtained with the 10 and 15 % (*v*/*v*) concentrations. As EG, glycerol had no effect on the NR fluorescence of either *S. obliquus* (Fig. 3g, h and i) or *C. pyrenoidosa* (Fig. 4g, h and i) in neither growth phase. In both growth phases of *Isochrysis* sp. (Fig. 5g, h and i), all the glycerol concentrations decreased the fluorescence.

### General remarks and guidelines

The NR staining of the *P. tricornutum* culture was successful with solvents. This could be seen in the difference of the fluorescence values obtained with and without solvents in the kinetic measurements. All the solvents increased and stabilized the fluorescence in the exponential and stationary growth phases, DMSO being the best. However, high solvent concentrations were needed, as also noted by Chen et al. (2009).

*C. pyrenoidosa* and *S. obliquus* stain poorly, which has been suspected to be due to rigid cell walls that NR is not able to penetrate (Chen et al. 2009). Chen et al. (2009) tested the effect of DMSO on several *Chlorella* species and found it to improve NR staining. Therefore, it was assumed that at least DMSO would increase the NR fluorescence of the green algae species of this study as well. However, none of the solvents increased the lipid-specific NR fluorescence of neither species in the stationary growth phase.

Huang et al. (2009) managed to successfully stain lyophilized *C. pyrenoidosa* cell powder dissolved in water with NR in DMSO. Chen et al. (2011) in turn further developed the method by Chen et al. (2009) by adding microwave heating to the DMSO treatment to facilitate staining of species with rigid cell walls. For the NR method to be applicable for routine monitoring purposes in microalgal cultivation, these additional steps introduce excessive complications and can be time consuming, e.g. the lyophilization taking 2 days (Huang et al. 2009). In this study, cells untreated prior to staining were therefore used, and penetration was not achieved with green algae species.

*Isochrysis* sp. cells stained best without any solvents or with very low concentrations of either DMSO or EG. All glycerol concentrations and the higher concentrations of DMSO and EG seemed only to harm the *Isochrysis* sp. cells by lowering the fluorescence.

In conclusion, the results show that NR staining times, the utility of solvents and optimal solvent concentrations cannot be standardized across different microalgal species. For some species, the NR method can be applied as a reliable proxy for neutral lipid accumulation without additional solvents, some species require a specific solvent to enhance the dye penetration, and for some species, the method is not applicable at all. As the NR method still offers unrivalled potential for near real-time monitoring of lipid accumulation in microalgal cultivation, the measurement kinetics and solvent effects should be carefully verified for each species of interest.
